# Investigating Effects of Bordered Pit Membrane Morphology and Properties on Plant Xylem Hydraulic Functions—A Case Study from 3D Reconstruction and Microflow Modelling of Pit Membranes in Angiosperm Xylem

**DOI:** 10.3390/plants9020231

**Published:** 2020-02-11

**Authors:** Shan Li, Jie Wang, Yafang Yin, Xin Li, Liping Deng, Xiaomei Jiang, Zhicheng Chen, Yujun Li

**Affiliations:** 1 Department of Wood Anatomy and Utilization, Research Institute of Wood Industry, Chinese Academy of Forestry, Beijing 100091, China; lishan@caf.ac.cn (S.L.); wangjie@caf.ac.cn (J.W.); yafang@caf.ac.cn (Y.Y.); dlp@icbr.ac.cn (L.D.); xiaomei@caf.ac.cn (X.J.); 2 Wood Collections (WOODPEDIA), Chinese Academy of Forestry, Beijing 100091, China; 3 College of Forestry, Beijing Forestry University, Beijing 100083, China; furyclaire@bjfu.edu.cn; 4 International Center for Bamboo and Rattan, Beijing 100102, China; 5 Institute of New Forestry Technology, Chinese Academy of Forestry, Beijing 100083, China; zcchen@caf.ac.cn; 6 School of Mechanical Engineering, Northwestern Polytechnical University, Xi’an 710072, China

**Keywords:** pit membranes, morphology, 3D reconstruction, microflow modelling, chemical composition, mechanical property

## Abstract

Pit membranes in between neighboring conduits of xylem play a crucial role in plant water transport. In this review, the morphological characteristics, chemical composition and mechanical properties of bordered pit membranes were summarized and linked with their functional roles in xylem hydraulics. The trade-off between xylem hydraulic efficiency and safety was closely related with morphology and properties of pit membranes, and xylem embolism resistance was also determined by the pit membrane morphology and properties. Besides, to further investigate the effects of bordered pit membranes morphology and properties on plant xylem hydraulic functions, here we modelled three-dimensional structure of bordered pit membranes by applying a deposition technique. Based on reconstructed 3D pit membrane structures, a virtual fibril network was generated to model the microflow pattern across inter-vessel pit membranes. Moreover, the mechanical behavior of intervessel pit membranes was estimated from a single microfibril’s mechanical property. Pit membranes morphology varied among different angiosperm and gymnosperm species. Our modelling work suggested that larger pores of pit membranes do not necessarily contribute to major flow rate across pit membranes; instead, the obstructed degree of flow pathway across the pit membranes plays a more important role. Our work provides useful information for studying the mechanism of microfluid flow transport across pit membranes and also sheds light on investigating the response of pit membranes both at normal and stressed conditions, thus improving our understanding on functional roles of pit membranes in xylem hydraulic function. Further work could be done to study the morphological and mechanical response of bordered pit membranes under different dehydrated conditions, as well as the related microflow behavior, based on our constructed model.

## 1. Introduction

Xylem sap is transported under negative pressure (tension) mainly driven by surface tension generated from transpiration at cell wall surfaces of leaf stomata [1]. The pulling force from numerous air-water menisci at leaf stomatal cell walls overcomes gravity and hydraulic resistances of xylem sap and produces the fluid tension within nm-scale capillary spaces of highly interconnected xylem conduits (vessels or tracheids which are typically 20 to 200 m in diameter [2]). Meanwhile, the hydrogen bond between water molecules also creates surface tension and adheres water to the hydrophilic cell wall, together ensuring continuous water transport in the xylem tissue [3]. In between neighboring conduits, thousands of bordered pits are distributed [4], which provide water conducting channels radially, axially and tangentially, accounting for 50% of the total xylem hydraulic resistance [5].

Since xylem sap is lifted up in a metastable state through bordered pits and could be interrupted by air bubbles in the conduits [6], embolism occurs in the xylem network and could be accelerated under certain environmental stress such as drought, frost, fungi invasion, etc., which further leads to xylem dysfunction and tree mortality [7,8,9]. The air-seeding hypothesis has been generally accepted as the principle mechanism for embolism formation [10,11], i.e., a tiny air bubble could pass through inter-conduit pit membranes from an embolized conduit into a neighboring functional conduit under certain pressure gradient [12]. Pit membranes, together with pit chambers, pit apertures as well as pit canals consist of bordered pits, which are overarching openings formed by uneven thickening of cell walls during conduit development. Bordered pits play important roles for plant hydraulics as they serve as crucial units in the xylem network for both water transport and air seeding [13,14]. A growing body of evidence has shown that inter-conduit bordered pit membrane is a key factor determining xylem embolism resistance [4,15,16] and further drought resistance [17,18]. Nevertheless, our knowledge on the pit membrane properties, such as its morphological characteristics especially in three-dimensional perspective, chemical compositions as well as mechanical properties is incomplete, which partly impedes our understanding about its functional role in plant hydraulics, as well as its response under certain environmental stress conditions. In this paper, a summary of pit membrane morphology and properties, their roles in plant hydraulics as well as the response of pit membranes under environmental challenges was demonstrated, which could potentially provide more detailed information for studying xylem hydraulics from individual perspective in microscopic and ultrastructural scale. Current debate on pit membrane properties such as chemical components of pit membranes was also discussed, and potential techniques or facility to solve future research questions were proposed. Besides, three-dimensional structure of bordered pit membranes was modelled. Furthermore, based on the reconstructed 3D pit membranes model, a virtual microfibril network was generated to model the microflow pattern across inter-vessel pit membranes. In addition, mechanical behavior of intervessel pit membranes was estimated from a single microfibril’s mechanical property. Our work might provide useful further information for studying water transport mechanism from pit membrane level, as well as the response of pit membranes both in normal condition and under certain stress levels.

## 2. Role of the Pit Membrane Morphology in Plant Hydraulics

The bordered pit membrane is composed of primary cell wall and middle lamella, with its structure varying greatly in angiosperm and gymnosperm tree species. In angiosperm tree species, bordered pit membrane is typically homogenous with pit annulus showing similar electron density under transmission electron microscopy [19]. Minute pores on pit membranes of angiosperm tree species allow water to pass through neighboring conduits and meanwhile induce embolism propagation. Nevertheless, the appearance of pit membranes for some angiosperm species differs from granular to smooth shapes [20,21], and its porosity could also be irregular, which might be induced from inherent developmental flaws or sample preparation artefacts [22]. In contrast, pit membrane of gymnosperm species is typically heterogeneous, especially in conifers [23]. The pit membrane of conifers consists of a central thickening pad generally impermeable and sometimes with punctures [24] and relatively thinner and highly porous margo, which is permeable to water and air bubbles, and meanwhile provides mechanical strength.

**Figure 1:**
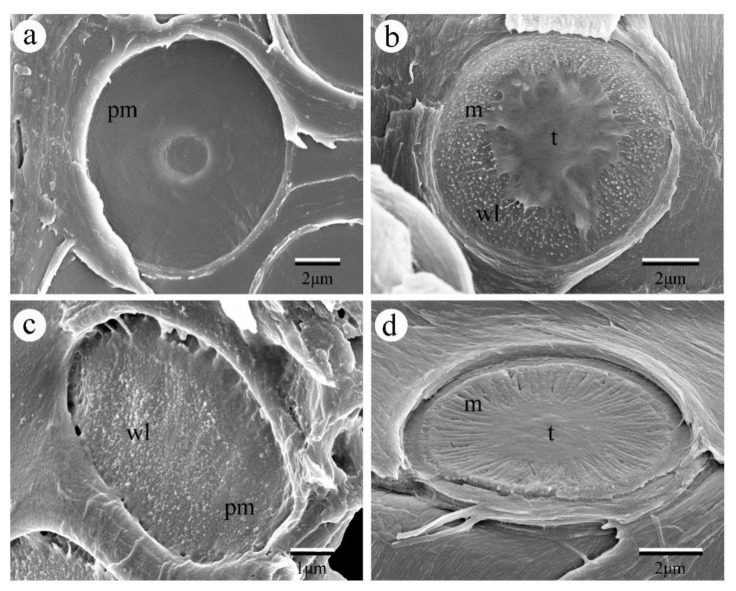
SEM images of pit membrane in between vessels of different angiosperm species and tracheids of gymnosperm species. (**a**) The pit membrane from *Catalpa bungei* C. A. Mey. showing very smooth surface with pit aperture visible through the pit membrane. (**b**) The pit membrane of *Cedrus deodara* (Roxb.) G. Don., showing scalloped torus margins and abundant warty layers inside the pit chamber. (**c**) The pit membrane from the vessel of *Salix matsudana var. pseudo-matsudana*, showing granular surface with many warty layers. (**d**) The pit membrane of *Cephalotaxus sinensis*, demonstrating extremely thick margo. Pm, pit membrane; m, margo; t. torus; wl. warty layer.

Here, we collected frozen wood samples from sapwood in the main truck of *Catalpa bungei,* fresh wood samples from 1–5-year-old stems of *Cedrus deodara, Salix matsudana var. pseudo-matsudana* and *Cephalotaxus sinensis*. Samples were split tangentially into small blocks (10 × 5 × 1 mm) and oven-dried at 60 ◦C or air-dried for 24 h. The blocks were subsequently coated with gold at 15 mA in anion sputter coater (Hitachi E-1045, Hitachi, Ltd., Tokyo, Japan) for one minute, and observed with an SEM (Hitachi S4800, Hitachi, Ltd., Tokyo, Japan) under high vacuum (5 kV accelerate voltage, ~15 mm working distance, se detector). Our SEM results show that pit membranes of the studied angiosperm tree species showed homogenous pit membranes while that of gymnosperm species showed heterogeneous pit membranes. Moreover, pit membrane morphology also differs among gymnosperm tree species, showing different torus thickness and margo stiffness (Figure 1), which may further determine their water transport efficiency.

### 2.1. Pit Membrane Thickness Variation and Its Hydraulic Functional Role

Within a wide range of angiosperm tree species, a more than 25-fold variation of pit membrane thickness was reported, with the mean intervessel pit membrane diameter around 10 μm and mean thickness around 300 nm [4,15]. Interspecific variation of pit membrane thickness might be due to genetic differences among tree species during cell wall formation [25,26]. In the vertical direction of tree individuals, the thinnest pit membrane was found in the largest vessel of sapwood along the main trunk in *Tocoyena formosa* and *Tabebuia aurea*; however, its thickness variation pattern was independent from tree height [27]. In contrast, the thickest pit membrane was detected at 4 m height aboveground with widest vessel of the sapwood along the trunk in *Eucalyptus grandis* [28]. Hence, whether larger vessels are more prone to show thinnest or thickest pit membrane appeared controversial, and the influence of tree height and cambial age on pit membrane thickness seemed to be species dependent. For instance, pit membrane thickness was found not to be affected by cambial age and axial sampling position in mangrove stem sapwood [29]. However, pit membrane thickness appeared to be thicker in the heartwood compared with sapwood of *Catalpa bungei* [30], while older growth rings tend to have shrunken and slightly thinner pit membranes in *Acer pseduoplatnus* [31]. Given that a strong positive correlation exists between xylem embolism resistance and pit membrane thickness [4], if larger vessels are more vulnerable to xylem embolism, one would expect larger vessels should have thinner pit membranes. Although there is convergent vessel widening from tree top to tree base [32,33], and vessel diameter tends to increase radially from the pith to the bark [30], no general agreement has been achieved on whether larger or smaller conduits are more vulnerable to xylem embolism [34], which might yield conflicting findings on pit membrane thickness from different studied tree species as mentioned above. Therefore, multiple tree species are needed to further explore the axial and radial variation of pit membrane thickness.

Within trees, pit membrane thickness varies across organs [31], which are possible explanations for within-tree variation xylem embolism resistance among different organs [35,36,37]. According to the xylem hydraulic segmentation hypothesis, distal parts of plants are more vulnerable to embolism [12], which was validated in many species [38,39,40,41] while no significant difference was observed in other species [42,43,44]. Specifically, roots are generally regarded more vulnerable to xylem embolism compared with stems [45], and the pit membrane of roots is thinner compared with stems [31,46]. This confirmed previous report that thicker pit membranes tend to be more resistant to xylem embolism compared with thinner pit membranes [4].

Besides interspecific and intraspecific variation of pit membrane thickness [31,47], its variation is also influenced by growing conditions [48], with species grown in drier habitat showing thicker pit membranes compared with those grown in humid areas [15]. Moreover, species grown in drier condition are more resistant to drought [49], suggesting that they have more embolism resistant xylem, which may be directly associated with their relatively thick pit membranes. In addition, in the same tree species, pit membrane thickness is significantly reduced under dehydrated conditions with shrinkage ratio about 50% [50], which might be due to the response of chemical composition of pit membranes as well as its mechanical properties from hydrated to dehydrated conditions.

### 2.2. Three Dimension of Intervessel Pit Membrane and Its Function

**Figure 2:**
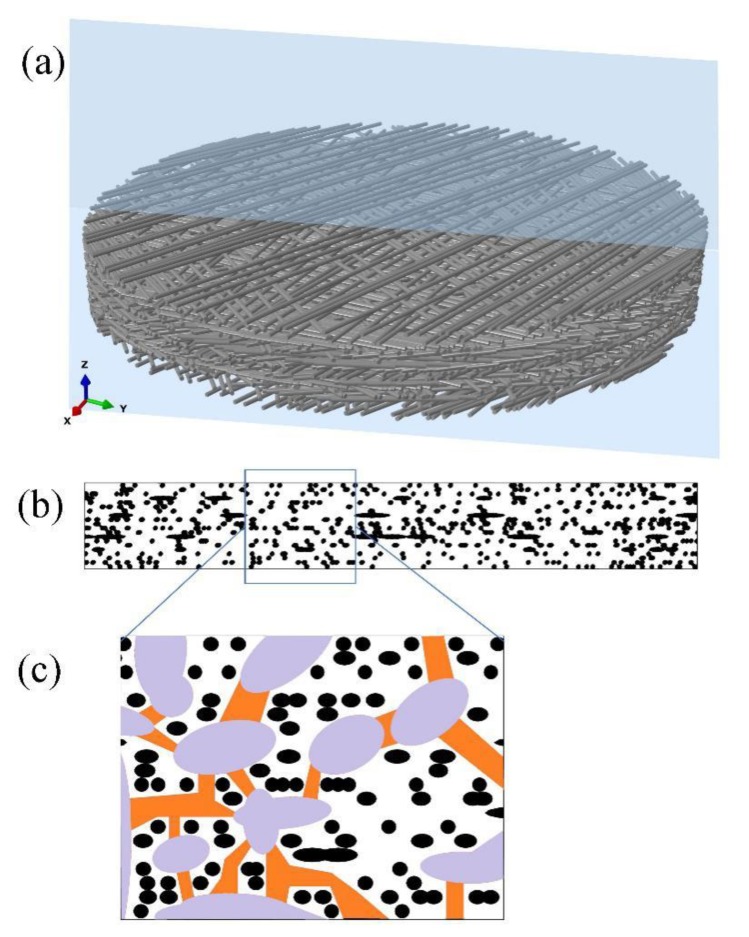
Three-dimensional reconstruction images of intervessel pit membrane. (**a**) A general view of intervessel pit membrane showing multiple layers of randomly distributed cellulose microfibrils. The blue slide shows direction of splitting in the radial view assuming pit membranes are circular. (**b**) Cellulose microfibril distribution of pit membrane in Figure 2a from the radial view, and black shapes represent cellulose microfibrils. (**c**) An enlarged view from the frame shown in Figure 2b, with pit membrane constriction illustrated. Black shapes represent cellulose microfibrils; purple shapes represent relatively larger pores in between cellulose microfibrils, and orange shapes represent constrictions between these relatively larger pores.

Pit membranes in angiosperm species are composed of numerous layers of cellulose microfibrils, of which showing abundant pores with different sizes and shapes. These pores would allow water flow in between water conducting cells through pit membranes and meanwhile function as “bubble generators” by inducing air bubbles to expand and further embolism occurrence [51]. On the other hand, surfactants such as phospholipids in xylem sap distribute and coat around pit membrane pores might lower surface tension, reduce bubble nucleation requirements and hence stabilize air bubbles to minimize embolism risk [52,53]. Given that pit membrane structure is three dimensional, it was recently suggested to use the term constriction size instead of pore size [54]. Although 3D reconstruction of bordered pits has been investigated by various techniques such as resin casting [55], silicone micromolding [56], ptychographic X-ray computed tomography [54], 4Pi and confocal laser scanning microscope [57], the 3D reconstruction of pit membranes demonstrating its porosity and tortuosity, in other words, distribution of constrictions, is so far lacking except for a few simplified models [58]. Hence, more precise modelling work on 3D reconstruction of pit membranes is demanded to further study its morphology and related hydraulic function. Here we used a deposition technique presented in [59] to numerically reconstruct 3D structure of pit membranes (Figure 2a), and its cellulose microfibril distribution (Figure 2b), as well as its constrictions (Figure 2c). During this generation procedure, the microfibrils fell one by one on an imaginary flat plate until they contacted the plate surface or other deposited microfibrils. The microfibril orientations and locations were assumed to be completely random to mimic the realistic ultrastructure of pit membranes. The plate gradually became covered with in-plane distributed microfibrils. The deposition program ran until reaching the target number of microfibrils. Here, the number of microfibrils to be deposited at each step and the microfibril deposition sequence can be adjusted to increase the microfibril volume fraction. After deposition, a circular network was numerically clipped from the initial generated geometry for further analysis, as shown in Figure 2a. In addition, further work could be done to adopt the finite element simulation to compress the fiber stack along the thickness direction to achieve morphological variation of pit membranes, such as changes of constrictions. This might be useful in understanding pit membranes’ functional implication in different dehydrated conditions.

Our work confirmed previous report that pit membrane constrictions are neither straight nor zigzagging [54,58], although the pores were highly connected (Figure 2c). In addition, recently developed automatic collector of ultrathin sections scanning electron microscopy (Auto CUTS-SEM) has axial resolution of 100 nm and horizontal resolution of 15 nm [60], which would be satisfying requirements of high resolution for 3D reconstruction of bordered pit membranes from plant materials and further test the modelled intervessel pit membrane structure.

Unlike angiosperm species with pore sizes of fresh pit membranes typically ranging from 5 to 20 nm [50,61], the pore sizes of gymnosperm species are at 0.1μm scale [23,62]. Much larger margo porosity overcomes higher hydraulic resistance of tracheids compared with long vessels in angiosperm tree species, and the torus-margo structure of pit membranes in gymnosperm tree species functions as safety valve during water transport [63,64], consequently leading to greater hydraulic safety margin of gymnosperm compared with angiosperm [65]. For angiosperm species, the rare pit hypothesis, i.e., the frequency of the leakiest pits is significantly correlated with xylem embolism resistance by air seeding pressure, has been confirmed in a number of species [66,67]. Therefore, increased pit membrane porosity in principle should make xylem more susceptible to embolism [68]; however, it did not get experimental support [69].Even though, pit aperture diameter must be taken into account when considering the whole-pit hydraulic resistance, if the pore diameter is much larger than the pit aperture, as much of the resistance would come from the pit aperture [70].

### 2.3. Microflow Simulation across Intervessel Pit Membrane and Its Implication

According to Hagen–Poiseuille equation, the water flow rate in conduits is proportional to the fourth power of conduit diameter [6]; larger vessels might have much thinner pit membranes, probably having larger number of larger pit membrane pores, which would enhance the hydraulic efficiency of conduits and also increase xylem hydraulic risk. Moreover, pit membrane thickness was negatively correlated with the maximum pit membrane pore sizes [15], which supports the speculation that thinner pit membranes from larger vessels would have more leaky pores and be more vulnerable to xylem embolism. To further investigate the role of pit membranes in the trade-off between xylem hydraulic efficiency and safety and given that pit membrane has three-dimensional structure [54], investigations could be done to link 3D pit membrane structure to pit hydraulic conductivity and resistance. While obtaining direct experimental evidence is challenging, the computational fluid dynamics (CFD) model provides a direct and robust approach to estimate microflow pattern across pit membranes. Valli et al. 2002 [71], Schulte et al. 2012 [72] and Schulte et al. 2015 [46] applied the CFD model based on a simplified 3D pit membrane structure; nevertheless, more precise modeling work on pit membrane level is demanded.

Here, we developed a more precise fluid flow simulation framework based on the aforementioned virtual 3D network of pit membranes. For simplicity, the microfibrils were assumed to be rigid in the current simulation, which means the fluid flow does not cause any change of pit membranes’ structure. The fluid flow within pit membranes could be generally described as the continuum medium and solved under a prescribed pressure gradient across the thickness of pit membranes. As mentioned in [72], viscous forces dominated the flow regime through intervessel pit membranes due to the slow steady state liquid flow. In other words, the particle-based Reynolds number was much less than 1. Hence, we could neglect the effect of liquid inertia and model the fluid flow by Stokes equation, $\nabla p\;=\;\mu \nabla^{2}V$, where p denotes the pressure, μ denotes the fluid dynamic viscosity, and V denotes the velocity vector. This expression together with the continuity condition, ∇V = 0, could provide all the necessary equations for flow simulation. In both expressions, the symbol ∇ represents a partial gradient operation with respect to the spatial variables, which means ∇p representing the local pressure gradient. Besides, the rough surface of microfibrils suggested that it appeared to more appropriate to apply no-slip wall condition on all the microfibrils’ surface rather than slip wall condition, although further evidence was required. Thereafter, the aforementioned equations and conditions were combined with the implementation of a finite element method in COMSOL Multiphysics software (Comsol Inc., Los Angeles, CA, USA) to simulate the liquid flow. It should be noted that the assumption of steady state flow implies that though-thickness capillary effects were not considered in the current simulation framework. The capillary effect should be considered in future simulation because, in reality, it might affect the applied flow-driving pressure gradient during microfibrils impregnation. The corresponding volumetric averaged flow rates along the thickness direction were then obtained according to the simulation results. Thereafter, the equivalent hydraulic permeability or resistance could be theoretically calculated, for example, using one-dimensional Darcy’s law. Consequently, values of theoretical hydraulic conductivity across intervessel pit membranes were generated.

**Figure 3:**
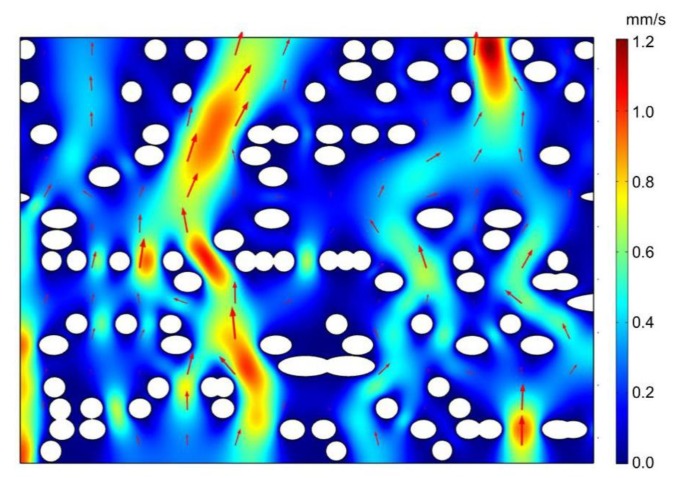
Flow velocity across intervessel pit membrane of angiosperm xylem from microfluid flow simulation. The fluid density was set to 998 kg/m^3^ with a dynamic viscosity, μ , of 10^−3^ Pa s, corresponding to the properties of water at 20 ℃. The pressure between the top and bottom surface was 1000 Pa in this example. White circles represent cellulose microfibril. Red region represents faster flow rate while blue region represents slower flow rate. Arrows represent velocity vector.

**Figure 4:**
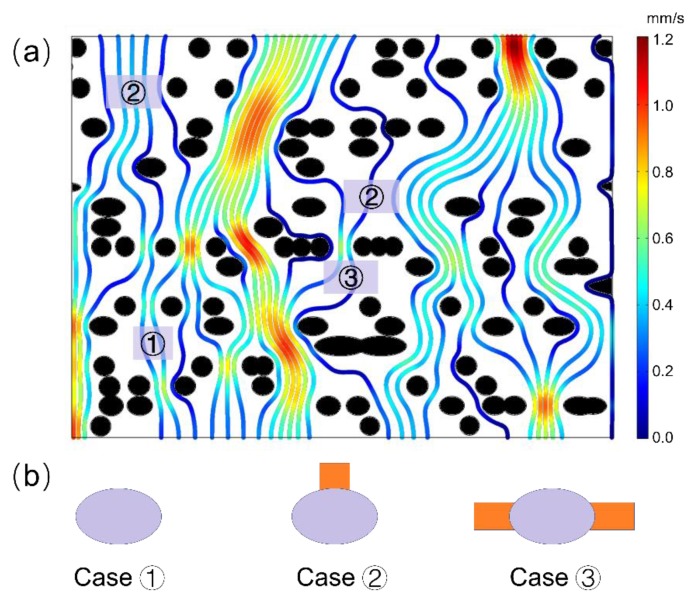
(**a**) Streamline diagram within intervessel pit membranes of angiosperm xylem from microfluid flow simulation. (**b**) Three typical obstructed cases with big pores hardly contribute to fluid transport, due to blockage of cellulose microfibrils in the direction across pit membrane thickness, i.e., fluid flow direction. In case 1, almost no water pathway was found around that pore, and in case 2, only one pathway was available, while in case 3, two pathways were available, however, contributing little flow rate. In Figure 4a, red color represents high flow rate while blue color represents low flow rate, and black circles represent cellulose microfibril. In Figure 4b, grey circles represent big pores while orange color represents water pathway.

The CFD simulation was also helpful to understand the fluid transport mechanism on the pit membrane level, for example, in angiosperm xylem. Based on the flow simulation from selected region of the 3D pit membrane structure (Figure 2b), we found that not all the large pores contributed to the major fluid flow within pit membranes (Figure 3). Only large pores without microfibrils along the fluid transport direction show high flow rate (Figure 4a). Other cases demonstrated only little flow rate even if the large pores have no obstacle in the other directions except the pit membrane thickness direction, i.e., flow path direction (Figure 4b). Therefore, the obstructed degree of flow pathway across the whole pit membrane thickness, i.e., the degree of microfibrils impeding the flow pathway, plays a more important role in fluid transport rather than the pore sizes.

### 2.4. Other Pit Membrane Related Traits and Functional Roles

In gymnosperm tree species, the quantitative characteristics of pits such as torus thickness and torus overlap (pit torus to aperture ratio) were strongly related to xylem embolism resistance [73]. Moreover, smaller pit aperture, less margo space (the distance between pit membrane border and torus relativized to pit membrane minus pit aperture diameter), together with torus overlap contribute to the trade-off between hydraulic efficiency and safety in ponderosa pine [70,74]. This would indicate that although disentangling the functional role of pit membranes in plant hydraulics, pit traits as a whole should be comprehensively considered when evaluating xylem hydraulic function, which might be essential for plant growth and mortality. In addition, the pit membranes show dynamic electron density during seasonal changes in current year stem of *Liridendron tulipifera*; however, pit membrane thickness was constant during different seasons [16]. Nevertheless, given that seasonal changes represent change of humidity, temperature and precipitation on the growing condition of plants, it is reasonable to propose that pit membrane thickness varies within different seasons from multiple tree species, which could partly explain seasonal variation of xylem embolism resistance [50]. Besides, previous work has shown that shading leads to thinner pit membranes [75], and drought also results in reduced pit membrane thickness (Li et al. unpublished data).

Additional pit traits such as pit membrane diameter are positively affected by cambial age, suggesting that larger pit membrane diameter in sapwood might improve hydraulic efficiency while smaller pit membrane diameter might be involved to effectively protect fungi invasion in heartwood [30]. The pit area hypothesis, i.e., larger pit area per vessel, was negatively correlated with xylem embolism resistance in many angiosperm species [76,77,78], however, not supported by [16,34,79], which again showed complexity in interpreting pit membrane traits due to diversity of studied tree species.

## 3. Role of Pit Membrane Chemical Composition in Plant Hydraulics

The main component of pit membranes is cellulose, which is embedded in the matrix of lignin, hemi-cellulose and pectin [29]. Hydrolase treatments showed that cellulose leads to sharp shift of xylem vulnerability curves, more vulnerable xylem and higher hydraulic conductance, while hydrolase for hemicellulose, proteinase, does not affect xylem hydraulic traits [80], which are indirect evidences for the cellulose as main components of pit membranes. Even though, the distribution of hydrophobic lignin and hydrophilic polysaccharides on xylem cell walls could influence xylem hydraulic functions [81]. Species with low xylem lignin content are more prone to xylem embolism, while species with high xylem lignin content varied with their embolism resistance [82]. Nevertheless, it is unclear if cellulose content is highly linked with xylem hydraulic efficiency and xylem embolism resistance from abundant tree species.

### 3.1. Chemical Components of Pit Membranes

Wood typically contains major chemical components such as polysaccharides (cellulose and hemicelluloses) and lignins, minor components, i.e., neutral solvent including soluble (fats, resin acid, essential oil) and insoluble (mineral, pectin and proteins) compounds [83]. As primary wood cell wall, generally accepted composition of pit membranes is to some degree clear, i.e., lignin, cellulose, hemi-cellulose and in some species pectin, although it is species specific. Lignin was found to be present in the pit membranes in some species [29,48,84,85], and different ratios of lignin monomers (S/G units) may modulate hydrophobicity and further hydraulic properties of the conduits [82,85]. The impregnation of lignin with microfibrils of pit membranes would reduce the wettability and thus lower air-seeding pressure, resulting in more embolism vulnerable xylem [82,85]. Whether hemi-cellulose was present in pit membranes especially in angiosperm species lacks wide investigation, partly due to its rather complex chemical structure [86]. However, it was suggested that hemicellulose is absent in pit membranes [85,87].

Distribution of pectin within pit membranes could be varied greatly in angiosperm and gymnosperm tree species. For example, in gymnosperm tree species, pectin is rich in the torus [88] while in mature pit membranes of angiosperm tree species it could be rare. Although pectin is not essential in pit membrane chemical composition, its presence appeared to be significantly associated with hydraulic efficiency. Homogalacturonans (HG), rhamnogalacturonan (RG)-I, RG II function as three main plolysaccharide domains, which account for ~65%, 20% to 35%, 10% of pectin, respectively [89]. As a major component of pectin, the methylation and demethylation pattern of HG could influence xylem hydraulics. During demethylation, Ca^2+^ bonds will be formed between pectin chains, resulting in swelling of pectin gels, which could increase hydraulic resistance of pit membranes [90]. Meanwhile, HG plays a crucial role in regulating deposition of cellulose microtubule [91], hence influencing initiation of pit membranes. Proteins distribute in pit membranes [92]; also, phenolic compounds and polyphenolic substances accumulate in pit membranes to defend it from pathogens [93,94]. Moreover, according to the nanoparticle hypothesis, i.e., lipids including phospholipids in xylem sap could coat onto pit membrane, stabilize air bubbles in xylem conduits and hence reduce the chance of embolism propagation [53,95]. Nevertheless, it remains an open question if lipids could penetrate pit membranes [53].

Chemical elements such as carbon, oxygen, potassium, calcium, chloride, phosphorus and sulfur are common in xylem sap; however, it is difficult to know about the chemical elements of pit membranes accurately as current techniques with high resolution are still limited. According to the transmission electron microscopy observation, the electron density of pit membranes during seasonal changes could be the variation of their chemical components [4,29]. Further work may be conducted by both qualifying and quantifying the chemical components and even chemical element variations of pit membranes during seasonal changes. A great challenge for doing such analysis could be resolution of current equipment such as confocal Raman microscopy. For instance, using fluorescent tagging and antibodies, the combination of confocal laser scanning Raman microscopy (CLSM) and 4Pi microscope could analyze molecular structure of fresh wood samples of *Pinus strobus* in situ [57], but the resolution is not satisfying. Recently, label-free Stimulated Raman Scattering (SRS) was reported to image chemical bonds with high sensitivity, resolution, speed and specificity [96], and have successfully quantified distribution of chemical components of plant cell wall such as lignin, suberin [97,98,99], thus being a potential tool to visualize the distribution and amount of chemical components in pit membranes in vivo with high resolution.

### 3.2. The Ionic Effect and Controversy of Pectin Presence in Pit Membrane

During water transport in xylem, the ionic effect, i.e., variation of cation concentration in xylem sap alters xylem hydraulic efficiency (no increase to >30%) compared with distilled water has been frequently reported in recent years [100,101,102,103,104,105,106]. It was suggested that electroviscosity is partially responsible for variation of hydraulic conductance of bordered pit membranes, which were reported to be attributed to shrinkage and swelling of hydrogel on the pit membranes [107,108,109] or pit membrane polymers instead of pectin [88]. Seasonal changes of ionic effect [110] might also be due to seasonal changes of pit membranes. In addition, the presence of Ca^2+^ decreased the K^+^ mediated enhancement of xylem hydraulic conductivity in hydrated stems [101,111]. Interestingly, the ionic effect did not substantially affect xylem vulnerability to embolism, while calcium is a major determinant for xylem embolism resistance [75,112], suggesting that the function of pit membranes in hydraulic flow and embolism resistance are uncoupled [101]. Moreover, it was suggested that ionic effect was not due to pit membrane properties [113], and its large variation was linked to vessel grouping indexes, or different parenchyma cells proportions and arrangement patterns, illustrating additional morphological traits to imply the mechanism of the ionic effect [102,114].

The existence of pectin in mature pit membranes of angiosperm species appears to be controversial [87]. Pectin was reported to be present in a few angiosperm species [110,115] or distributed only in the annulus of interconduit pit membranes [75,85,95] while completely absent in some studies [116]. By combining synchrotron infrared nanospectroscopy (SINS) and atomic force microscopy-infrared nanospectroscopy (AFM-IR) with high spatial resolution, recent work has shown that cellulose, phenolic compounds and proteins were found in intervessel pit membranes of *P. nigra*, but neither was pectin nor lignin [92]. Nevertheless, wood samples had to be completely dehydrated during measurements, which may lead to degradation of some chemical components. In addition, the absence of lignin in *P. nigra* does not exclude the lignification of pit membrane [81]. Therefore, although pectin presence depends on the studied species [87], applying SRS to observe chemical compositions pattern during different ontogeny stages of pit membranes might provide a fast way to investigate pectin present among multiple species.

## 4. Role of Pit Membrane Mechanical Properties in Plant Hydraulics

As anisotropic biomaterial, the mechanical property of bordered pit membranes [47,73] has received much more attention in plant hydraulics. The deformation response of pit membranes under different strain, as well as the strength and stiffness of homogenous and heterogenous pit membranes, including the pit torus and margo, could provide more evidence to validate the air seeding hypothesis. Under increasing tension, the deformation of pit membranes undergoes elastic and plastic stages [2], which requires precise measurements at higher magnification.

### 4.1. Techniques in Characterizing Mechanical Properties of Pit Membrane

In recent years, mesomechanical characterization was applied to the study of cell wall mechanics [19], and mechanical properties of pit membranes were also brought into focus especially due to their special structure and essential role in plant hydraulics. For instance, atomic force microscope with nanometer resolution combined with nanoindentation was applied to estimate the mechanical properties of pit membranes, showing that the Young’s modulus values of both wet and dehydrated pit membrane in hybrid poplar were similar [56]. However, given that the main chemical composition of pit membrane is hydrophilic and moisture sensitive cellulose microfibrils, interaction with water molecules between the cellulose polymer chains could initiate the breaking of interchain hydrogen bonds [117]. Consequently, the decrease in the amount of intact hydrogen bonds should in principle weaken the mechanical properties of pit membranes, as indicated by the molecular dynamics simulations. Therefore, lower elastic modulus values would be expected from the wet pit membranes compared with the dry ones, which could be tested by studying more species.

By applying force-displacement on pit membranes of conifers with quartz probes, the manipulator experiments show that pit membrane failure occurs from ruptured torus under increasing loading strain, i.e., torus prolapse. Although it is unlikely to happen in situ due to different surface of plant areas under pressure, the force leading to pit aspiration agreed with previous calculations on air seeding pressure by hydraulic measurements [118]. Therefore, mesomechanical tests would be indirect but useful tools to study pit membrane mechanical properties. Nevertheless, quantification work of mesomechanical properties of bordered pit membranes is relatively less, and higher measurement accuracy of the previously mentioned equipment is demanded in future work. Moreover, the movement of cell walls with pit membranes during mechanical measurements resulted in difficulty of directly obtaining pit membrane stiffness with previously mentioned techniques. Therefore, techniques with higher accuracy which could perform force-displacement measurement in situ might be helpful to overcome the shortcomings. Recently, in situ mechanical test of loading and unloading was successfully conducted in metal by focused ion beam-nanoindentation [98], which could be further applied for improving measurement accuracy of pit membranes.

### 4.2. Modelling Mechanical Properties of Pit Membrane

**Figure 5:**
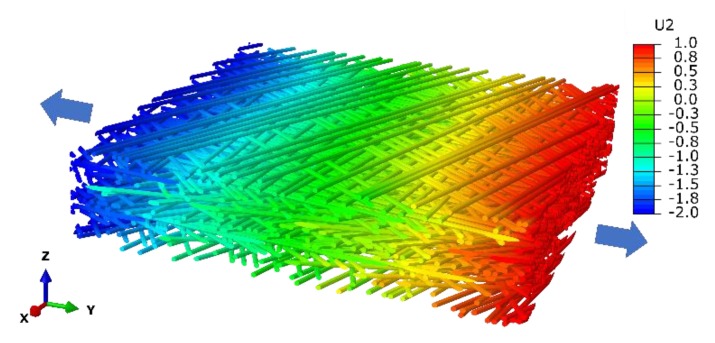
Finite element simulation of uniaxial tension on the microfibril network with displacement field (U2, nm) along the tension direction (Y axis). The legend illustrates values of different colors.

Besides experimental techniques to study mechanical behavior of pit membranes, theoretical calculation or numerical micromechanical methods provides alternative ways to further investigate its mechanical properties. Assuming the pit membrane as an ideal cellulose microfibril network consisting of long straight microfibrils oriented at random distribution, applying the well-known Cox’s model can directly correlate the microfibril property variation to the effective stiffness of the pit membranes [119], $E\;=\;v_{f}E_{f}/3$, where E and $E_{f}$ denote the Young’s modulus of pit membranes and microfibril, respectively, and $v_{f}$ denotes the volume fraction of microfibril in pit membranes. The simple formula reveals the dependence of the linear elastic property of pit membranes on its porosity volume and the individual microfibril’s property, thus further simulating the mechanical response of pit membranes under mild and moderate stress conditions, such as short-term exposure to dehydration. In order to quantify the nonlinear mechanical behavior of pit membranes, e.g., irreversible deformation and fracture of pit membranes, here we conducted the direct tension finite element simulation on the aforementioned virtual microfibril network, e.g., pit membrane model (Figure 5). The simulation we applied was identical to the practical uniaxial tensile experiment, which has been widely adopted to measure the mechanical properties of materials. This modeling framework enables us to evaluate the influence of moisture content on the effective stiffness and strength of pit membranes quantitively. It also provides insight into obtaining the detailed deformation at the microscale level, such as strength statistics and fracture mechanisms, to help understand mechanical response of pit membranes under several stress condition, such as extreme drought and frost, etc. Moreover, to simulate mechanical deformation of pit membranes under tension by water flow, our results from uniaxial tension simulation could be used in the uniform stretching (i.e., tensions in all directions) analysis reasonably.

### 4.3. The Reversibility of Pit Membranes under Environmental Changes

It is generally assumed that pit aspiration is reversible in living trees, and previous work has indirectly shown that freeze-thaw induced xylem embolism is reversible by xylem embolism repair, i.e., refilling, and so is pit aspiration [120]. Drought is one of the main factors inducing embolism, and under intensive and continuous drought conditions, an increase of xylem tension contributes to a higher-pressure gradient between a functional and non-functional vessel, which further increases the likelihood of xylem embolism and leads to a large number of dysfunctional vessels. Nevertheless, whether pit aspiration could be reversible was not examined with intact plants; also, response of pit membranes under environmental challenges, especially quantification of its geometrical characteristics under dehydration-rehydration events, such as drought-rewatering cycles and freezing thaw cycles in situ is surprisingly lacking.

It would be interesting to observe the deformation of pit membranes under drought conditions and combine it with the modelling work of pit membrane mechanical properties, such as its force-displacement progress under different drought treatments. Moreover, it would be more interesting to observe the reversal of pit membranes in vivo under drought-rewatering conditions. This would require non-destructive equipment with high resolutions, i.e., hundreds of nanometers [121] to detect the deformation details and reversal progress, which might lend solid support to in vivo validation of the reversal of pit membrane aspiration and xylem embolism repair.

## 5. Conclusions

In conclusion, the trade-off between xylem hydraulic efficiency and safety was closely related with properties of pit membranes, and xylem embolism resistance was also determined by the pit membrane properties. More precise equipment for quantifying and qualifying pit membrane properties is demanded to solve current debate and explore unknown features of pit membrane properties, such as chemical components of pit membranes and their reversibility under dehydration and rehydration cycles. Larger pores of pit membranes do not necessarily contribute to major flow rate across pit membranes; instead, the obstructed degree of flow pathway across the pit membranes plays a more important role. The reconstructed 3D structure of pit membranes, and modeling work on its mechanical property as well as microflow behavior might provide useful information for studying the function of pit membranes in plant hydraulics, as well as the response of pit membranes under external stress conditions.
